# Crude fucoidan content in two North Atlantic kelp species, *Saccharina latissima* and *Laminaria digitata*—seasonal variation and impact of environmental factors

**DOI:** 10.1007/s10811-017-1204-5

**Published:** 2017-07-05

**Authors:** Annette Bruhn, Tina Janicek, Dirk Manns, Mette Møller Nielsen, Thorsten Johannes Skovbjerg Balsby, Anne S. Meyer, Michael Bo Rasmussen, Xiaoru Hou, Bodo Saake, Cordula Göke, Anne Belinda Bjerre

**Affiliations:** 1 Department of Bioscience, Aarhus University, Vejlsøvej 25, 8600 Silkeborg, Denmark; 2 Department of Chemical and Biochemical Engineering, Technical University of Denmark, Søltofts Plads, 2800 Kgs. Lyngby, Denmark; 3 National Institute of Aquatic Resources, Technical University of Denmark, Dansk Skaldyrcenter, Øroddevej 80, 7900 Nykøbing Mors, Denmark; 4 Section for Biomass and Biorefinery, Danish Technological Institute, Gregersensvej 1, 2630 Taastrup, Denmark; 5 Department of Chemical Wood Technology, University of Hamburg, Hamburg, Germany; 6 Department of Bioscience, Aarhus University, Frederiksborgvej 399, 4000 Roskilde, Denmark

**Keywords:** Cultivation, Ecotypes, Exposure, Irradiance, Laminariales, Nutrients, Salinity, Storage carbohydrates

## Abstract

Fucoidans are sulphated fucose-rich polysaccharides predominantly found in the cell walls of brown algae. The bioactive properties of fucoidans attract increasing interest from the medico-pharmaceutical industries and may drive an increase in demand of brown algae biomass. In nature, the biochemical composition of brown algae displays a seasonal fluctuation driven by environmental factors and endogenous rhythms. To cultivate and harvest kelps with high yields of fucoidans, knowledge is needed on seasonal variation and impact of environmental conditions on the fucoidan content of brown algae. The relations between the fucoidan content and key environmental factors (irradiance, nutrient availability, salinity and exposure) were examined by sampling natural populations of the common North Atlantic kelps, *Saccharina latissima* and *Laminaria digitata*, over a full year at Hanstholm in the North Sea and Aarhus in the Kattegat. In addition, laboratory experiments were carried out isolating the effects of the single factors. The results demonstrated that (1) seasonal variation alters the fucoidan content by a factor of 2–2.6; (2) interspecific differences exist in the concentrations of crude fucoidan (% of dry matter): *L. digitata* (11%) > *S. latissima* (6%); and (3) the effects of single environmental factors were not consistent between species or between different conspecific populations. The ambiguous response to single environmental factors complicates prospective directions for manipulating an increased content of fucoidan in a cultivation scenario and emphasizes the need for knowledge on performance of local kelp ecotypes.

**Electronic supplementary material:**

The online version of this article (doi:10.1007/s10811-017-1204-5) contains supplementary material, which is available to authorized users.

## Introduction

Fucoidans are complex heterogeneous sulphated fucose-rich polysaccharides with various documented bioactive functions requested by the pharmaceutical, nutraceutical, cosmeceutical and functional food industries (Wijesinghe and Jeon 2012). They are typically structured around a backbone of α-linked L-fucose residues having various substitutions and containing, in addition to fucose, varying smaller proportions of other monosaccharides (galactose, mannose, xylose, rhamnose, glucose) and sugar acids (glucuronic acid) (Kloareg and Quatrano 1988; Li et al. 2008; Ale et al. 2011; Ale and Meyer 2013). Fucoidans are found in the cell walls of brown algae (Percival 1979). The documented bioactivities of fucoidans include antioxidant, anticancer, anticoagulant, antithrombotic, immunomodulant and antiproliferative effects (Holdt and Kraan 2011; Wijesekara et al. 2011). Over the recent years, the scientific and industrial interest in fucoidans has increased considerably (Ale et al. 2011), and fucoidans are often mentioned as key value-added compounds with a potential to improve the profitability of brown algae biorefineries (Cardoso et al. 2014; Lorbeer et al. 2015; Yuan and Macquarrie 2015).

The tissue concentrations as well as the specific structure and bioactivity of fucoidans vary within and between brown algae species, and a broad selection of methods for extraction and characterization adds to the variation found in the literature (Berteau and Mulloy 2003; Ale et al. 2011). The specific physiological functions of fucoidans within the algae cells are not well known (Kraan 2012). In kelps (Laminariales), the tissue concentration of fucoidans is suggested to be regulated by a number of factors, such as reproduction (Usov et al. 2005; Skriptsova et al. 2010, 2012; Vishchuk et al. 2012; Mak et al. 2013), part of thallus (Black 1954; Usov et al. 2005), age of plant (Zvyagintseva et al. 2003), season (Black 1954; Mak et al. 2013; Ehrig and Alban 2015; Skriptsova 2016) and environmental factors (Ehrig and Alban 2015; Skriptsova 2016) (Table 1). Fucoidan contents of kelp species are reported in the range of 0.5–13% of dry matter (DM) in sterile tissue and 1.4–69% in reproductive tissue (Table 1). The highest reported tissue content of fucoidans reported in any brown algae species (69% of DM) was from the reproductive tissue (sporophylls) of *Undaria pinnitifida* (Mak et al. 2013). Sporophylls of *Alaria* also have higher fucoidan content than non-reproductive tissue (Usov et al. 2005; Vishchuk et al. 2012), and even in Laminaria species without distinct sporophylls, the reproductive tissue (sori) contains more fucoidan than non-reproductive tissue (Vishchuk et al. 2012). In the non-reproductive tissue, fronds contain more fucoidan than stipes and midribs (Black 1954; Usov et al. 2005), and older plants tend to contain more fucoidan than young plants (Zvyagintseva et al. 2003).

**Table 1:** Overview of published fucoidan content in Laminariales and suggested factors with impact on tissue fucoidan content: *E* environment, *R* reproduction, *T* part of thallus, *S* season, *A* age of plant, *Sporo* sporophyll

Species	Part of thallus	Country	Sea	Content (% of DM)	Impact	Reference
*Alaria fistulosa*	Frond	Russia	OS	0.7	T	Usov et al. (2005)
	Midrib			0.6		
	Sporo			7.8		
	Stipes			0.5		
*Alaria ochotensis*	Sporo	Russia	OS	3.4–7.0	R	Skriptsova et al. (2012)
*Alaria* sp.	Frond	Russia	OS	3.8	R	Vishchuk et al. (2012)
	Sporo			5.7		
*Ecklonia radiata*	Whole plant	Australia	SO	2.3–3.2		Lorbeer et al. (2015)
	Whole plant	Australia	SO	3–3.7		Charoensiddhi et al. (2016)
*Laminaria claustonii*	Stipes	England	NA	1.8–3.4	T S	Black (1954)
	Frond		NA	2.4-4.5	T S	
*Laminaria digitata*	Frond	England	NA	2.2–3.8	S	
	Frond	France	NA	3.5		Mabeau et al. (1990)
				5.5		MacArtain et al. (2007)
	Frond	Denmark	NS	3.4–11.2	S (E) (R)	This study
			BA	2.8–7.4	E S	
*Saccharina cichorioides* (*Laminaria cichorioides*)	–	Russia	SJ	3.5–7.8	S A	Zvyagintseva et al. (2003)
*Saccharina japonica* (*Laminaria japonica*)	Frond	Japan	PO	2.2–4.3	S	Honya et al. (1999)
	–	Russia	SJ	2.4–2.9	A	Zvyagintseva et al. (2003)
				1.7		Mizuno et al. (2009)
	Whole plant	Russia	OS	6–8	R	Skriptsova et al. (2012)
	Frond	Russia	OS	0.7	R	Vishchuk et al. (2012)
	Sori			1.4		
	Whole plant	Russia	SJ	0.9–4.3	E S A	Skriptsova (2016)
	Sori			2.6–3.4	A R	
*Saccharina latissima* (*Laminaria saccharina*)	Frond	England	NA	2.1–2.7	S	Black (1954)
	Frond	Russia	BS	8.8		Obluchinskaya (2008)
	Frond	Faroe Islands	NA	3.8–4.2	E S	Ehrig and Alban (2015)
	Frond	Germany	BA	1.8–2.3	E S	
	Frond	Denmark	BA	2.3–6.2	E S	This study
*Saccharina longicruris* (*Laminaria longicruris*)	Frond	Canada	NA	1.8–4.5	S	Rioux et al. (2009)
*Saccharina longissima* (*Laminaria angustata* var*. longissima*)	Frond	Japan	PO	2.6		Kitamura et al. (1991)
*Undaria pinnitifida*	Whole plant	Russia	SJ	3.2–16.0	R S	Skriptsova et al. (2010)
	Frond	New Zealand	SPO	3–13	R S	Mak et al. (2013)
	Sporo			25–69	R S	

The different seas are abbreviated as follows: *OS* Okotsk Sea, *SO* Southern Ocean, *NA* North Atlantic, *NS* North Sea, *BA* Baltic Sea, *SJ* Sea of Japan, *PO* Pacific Ocean, *BS* Barent Sea, *SPO* South Pacific Ocean

As with other tissue components, the relative tissue content of fucoidans varies over the year (Black 1954; Honya et al. 1999; Rioux et al. 2009; Skriptsova et al. 2010; Mak et al. 2013; Ehrig and Alban 2015; Skriptsova 2016). Generally, an increase over the year from March/April to July/September was described on the northern as well as the southern hemisphere: a peak in fucoidan content in spring (September) was described for *U. pinnitifida* in New Zealand (Mak et al. 2013), while spring (March/April) was reported as the periods of minimum and summer/autumn (July–October) reported as the period of maximum contents of fucoidan in North Atlantic, Japanese and Russian Laminariales (Black 1954; Honya et al. 1999; Obluchinskaya 2008; Skriptsova et al. 2010; Ehrig and Alban 2015; Skriptsova 2016). In some cases, the increase in fucoidan coincided with the maturation of reproductive tissue (Mak et al. 2013; Skriptsova et al. 2010, 2012; Skriptsova 2016). Based on these studies, it has been suggested to optimize the harvest yield of fucoidan through optimizing time of harvest (Ehrig and Alban 2015). Optimizing the yield of fucoidans from cultivated or harvested kelps requires fundamental knowledge of the natural seasonal variation in tissue fucoidan content, comprising the effects of the underlying environmental factors triggering the variation. Environmental and endogenous factors determine the seasonal cycle of growth and reproduction of perennial algae such as the Laminariales (Bartsch et al. 2008), hereby also temporal and spatial fluctuations in tissue contents of storage carbohydrates (laminarin and mannitol) are influenced (Manns et al. 2017). Thus, the effects of environmental conditions on the relative tissue content of fucoidans could be regulated both directly and indirectly. Only limited knowledge is published on the direct effect of single environmental factors on the regulation of the tissue content of this interesting group of polysaccharides. A positive effect of salinity on the fucoidan content of *Saccharina latissima* was suggested (Ehrig and Alban 2015), whereas for *Saccharina japonica*, the fucoidan content was documented to correlate positively with temperature and negatively with environmental nitrate concentrations (Skriptsova 2016). Fucoidans appear to stabilize the cell wall by cross-linking between matrix cellulose microfibrils and thereby strengthening the cell wall (Deniaud-Bouet et al. 2014). Thus, a putative function of protection against mechanical, chemical and osmotic stress to the cell wall is supported by key environmental factors being coupled directly to the tissue contents of fucoidan: (1) degree of exposure (mechanical stress): Black (1954) showed that fronds of *Laminaria digitata* and *Laminaria cloustonii* from more protected lochs had a higher L-fucose contents than open sea fronds (Black 1954) indicating a negative effect of exposure on the tissue content of fucoidan; (2) salinity (osmotic stress): individuals of *S. latissima* in the saline North Atlantic were shown to have higher fucoidan content than individuals of the same species in the more brackish Baltic Sea (Ehrig and Alban 2015); and finally (3) high light, UV radiation and free radicals stimulated by fluctuations in environmental factors were argued, but not shown, to stimulate higher tissue contents of fucoidan in brown algae, due to the antioxidant protective function in the brown algae cell wall (Holtkamp 2009).

Indirectly, environmental factors could contribute to seasonal fluctuations in the relative tissue content of cell wall carbohydrates, including fucoidans, as cells enlarge to accommodate larger volumes of storage carbohydrates over summer when nitrogen deprivation limits the cell division (Black 1950; Nielsen et al. 2016a). As the cells accumulate storage carbohydrates, the cell walls potentially become relatively thinner due to “stretching”, or even if the cell wall composition and thickness is unaltered, the relative tissue content of cell wall carbohydrates would decrease as the volume increases, as a mere consequence of the surface to volume ratio of a sphere.

With the perspective of guiding commercial kelp cultivation towards higher yields of fucoidans, the aims of this study were to describe the seasonal variation in fucoidan tissue content and evaluate the direct and indirect impacts of key environmental factors on the content of crude fucoidan of two common, and commonly cultivated, North Atlantic kelp species: *S. latissima* and *L. digitata*. Natural populations at two locations, Hanstholm at the North Sea coast and Aarhus at the Baltic Kattegat coast, were sampled during a full year, and contents of crude fucoidan were related to large-scale environmental factors (salinity, exposure and availability of nutrients and light), as well as to the tissue carbohydrate composition. Further, the effects of isolated factors (salinity, light and nutrient concentrations) on the content of crude fucoidan were tested under controlled conditions.

## Materials and methods

### Seasonal variation in environmental factors

**Fig. 1:**
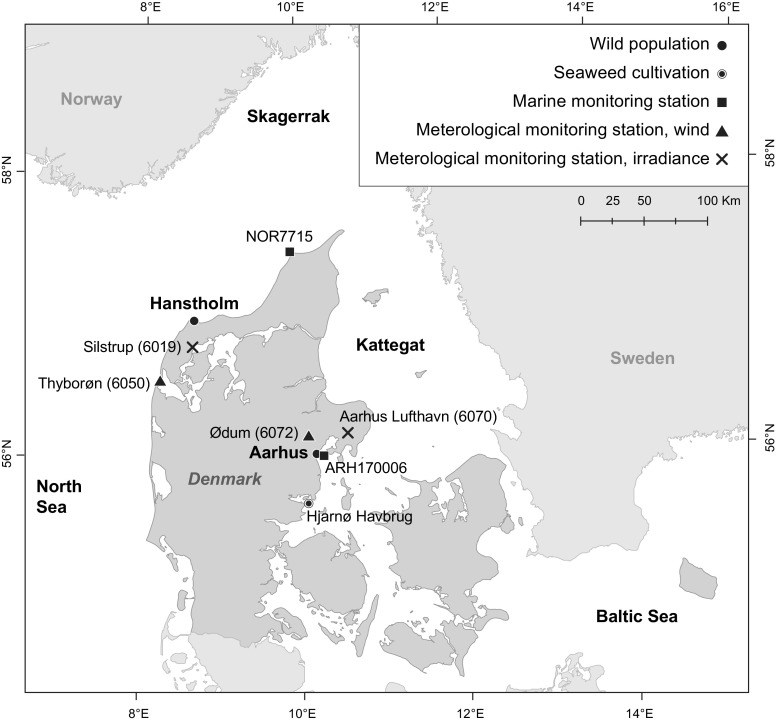
Map of Denmark showing the locations of the populations of kelp sampled for determination of seasonal variation (Hanstholm and Aarhus) and for experimental work (Aarhus and Hjarnø Havbrug). Marine environmental monitoring stations from which data was supplied on salinity, temperature and nutrient concentrations are indicated as *black squares*. Meteorological monitoring stations from which data was supplied on wind and irradiance are indicated as *black triangles* and *crosses*, respectively

Data on seawater salinity, nutrient concentrations and temperature were extracted from the National Database for Marine Data (MADS) hosting all monitoring data of the Danish National Aquatic Monitoring and Assessment Program (DNAMAP). Data were extracted from the monitoring stations with closest proximity to each of the two sampling locations. For the Hanstholm population, data from station NOR7715 was used (distance to sampling location: 85 km). For the Aarhus population, data from station ARH170006 was used (distance to sampling location: 6.5 km) (Fig. 1). At the environmental monitoring stations, sampling for seawater quality analyses was performed 4–6 times per month at a depth of 1 m. Sampling and the following analyses of seawater quality was performed using standard methods according to the Danish marine monitoring programme (NOVANA) (Markager and Ærtebjerg 2004).

Data on accumulated daily surface irradiance, wind velocity and wind direction were obtained from The Danish Meteorological Institute, from the following stations: wind—Hanstholm: Thyborøn 6052, Aarhus: Aarhus lufthavn 6070; and irradiance—Hanstholm: Silstrup 6019, Aarhus: Ødum 6072 (Vilic 2013) (Fig. 1). Irradiance is given as daily averages of μmol photons m^−2^ s^−1^.

Following Fonseca et al. (2002), who modified the equation for the relative exposure index (REI) from Keddy (1982), the REI was calculated for each sampling location based on data on wind direction and velocity as:

$$ \mathrm{REI}=\sum_{i=1}^8\left({F}_i\ \mathrm{x}\ {W}_i\ \mathrm{x}\ {P}_i\right) $$

where *F*_*i*_ is the effective fetch (m) in the direction *i* (N, NE, E, SE, S, SW, W, NW) from which the wind with the average wind velocity *W*_*i*_ (m s^−1^) for 14 days prior to the collection of the seaweed samples for the percentage of time (*P*_*i*_) is approaching. The effective fetch was calculated as the weighted mean of the distance of the sample point to land (with a maximum length of 10 km) in the direction *i* and in 4 neighbouring directions with 11.25° distances, weighing by multiplying with the cosine of the resulting angle.

### Sampling and pre-treatment of seaweed

#### Sampling for analysis of seasonal variation in natural populations

The algae material for the analysis of seasonal variation of crude fucoidan content was sampled with monthly to bimonthly intervals in 2012 and 2013 at two different locations in Denmark: at Hanstholm, located at the Danish North Sea coast (57° 07′ 10″ N, 08° 39′ 14″ E), and at Aarhus, at the Danish coast of Kattegat (the Baltic Sea) (56° 10′ 09″ N, 10° 13′ 36″ E) (Fig. 1). *Laminaria digitata* (Hudson) J.V. Lamouroux was sampled at both locations. *Saccharina latissima* (Linnaeus) C.E. Lane, C. Mayes, L. Druehl & G.W. Saunders was only sampled at Aarhus, since no *S. latissima* was present at or near Hanstholm. A minimum of ten intact adult sporophytes were sampled at each location from 1 to 3 m depth by snorkelling. The sporophytes were approximately 2–3 years old, estimated from the size and morphology of frond and stipes. Sampling at Hanstholm was performed on the following dates: in 2012: 29 Aug. 28 Nov; and in 2013: 18 Jan. 07 Mar, 02 Apr, 21 May, 01 Jul, 27 Aug and 28 Nov. Sampling at Aarhus was performed in 2012: 16 Oct, 29 Nov; and in 2013: 24 Jan, 20 Feb, 22 Mar, 25 Apr, 24 May, 28 Jun, 17 Jul, 20 Aug, 04 Sep and 24 Oct. Reproductive status was recorded in the Aarhus populations from March 2013 and onwards as presence/absence of sori in the sampled sporophytes (% fertile sporophytes of total number sampled). Reproductive status was not consistently recorded in samples from the Hanstholm population, and only one note from January 2013 described the reproductive status of this population. Samples were transported to the lab and initially frozen at −20 °C, then freeze dried at −40 °C, homogenized by dry milling and kept at −20 °C until further analysis.

#### Sampling for experimental work

*Laminaria digitata* for the experimental work was harvested by snorkelers in June 2015 from wild populations at Aarhus Bay (56° 10′ 8.74″ N, 10° 13′ 35.55″ E). The *L. digitata* sporophytes for the experimental work were approximately 2 years old. The samples of *S. latissima* for the experimental work were delivered in January 2015 from a commercial aquaculture production at Hjarnø Havbrug A/S (55° 48′ 33.97″ N, 10° 07′ 01.40″ E) (Fig. 1). The *S. latissima* sporophytes for the experimental work were approximately 1.5 years old. Seaweed samples were kept in plastic bags during transportation to the lab.

In the lab, the seaweeds were stored for 2 days in aerated artificial sea water (ASW) (Red Sea Coral Pro Salt, Red Sea Fish Pharm Ltd., Israel) at a salinity of 25, 10 °C, 100 μmol photons m^−2^ s^−1^ and enriched with f/2 medium (to a final concentration of 884 μM NO_3_^−^ and 36 μM PO_4_^3−^) (Guillard and Ryther 1962).

### Experimental work

Two laboratory experiments were carried out with the aim of testing the effects of isolated environmental factors on the biochemical composition of the two kelp species. The experiments were performed in glass beakers each containing 2 L of ASW added with vitamins and trace metals as for f/2 medium (Guillard and Ryther 1962). The cultivation conditions were set as recommended by Andersen et al. (2013): temperature was kept at 10.7 ± 1.5 °C (measured once a day), with a light:dark cycle of 16:8. The light was supplied from above the beakers using cool fluorescent white light tubes (50/50 combination of Philips Master TL5 HO 39 W/840 and Philips Master TL5 HO 39 W/830). The specific level of irradiance for each individual experiment is described below. All experiments were carried out over 14 days, during which the water was exchanged every second day. All beakers were continuously aerated with atmospheric air.

The start fresh weight (FW) of each of the algae was between 30 and 50 g, the length of the fronds ranged between 25 and 35 cm. The FW of each frond was measured before and after the experiments. At the end of the experiments, the algae were frozen at −20 °C, and subsequently freeze dried at −40 °C for 24 h. The dry weight (DW) of the dried algae material was determined and the material was dry milled to a fine powder for further analysis.

#### Experiment 1—salinity

A total of 9 young intact fronds of *S. latissima* were kept separately in 9 beakers as triplicates of three salinity treatments (10, 20 and 30). The irradiance was kept at 45–70 μmol photons m^−2^ s^−1^. Throughout the experiment, all beakers received the same amount of light, as they were moved around every day. The nutrient concentrations were 1/100 of f/2 (8.84 μmol NO_3_^−^-N and 0.362 μmol ortho-P).

#### Experiment 2—light and nutrients

A total of 18 young intact fronds of *L. digitata* were kept separately in beakers as triplicates of a total of 6 experimental conditions: two different light intensities—non-saturating light (15–35 μmol photons m^−2^ s^−1^) and saturating light (100–125 μmol photons m^−2^ s^−1^), respectively (Middelboe et al. 2006; Bartsch et al. 2008), and three nutrient concentrations, chosen to reflect the range of natural nutrient availabilities in Danish waters—low, 0 μM NO_3_^−^-N and 0 μM ortho-P; intermediate, 8.84 μM NO_3_^−^-N and 0.36 μM ortho-P; and high, 44.2 μM NO_3_^−^-N and 3.6 μM ortho-P (Hansen 2015). The irradiance was measured every day during the experiment, using a Li-Cor, Li-1000. The pH fluctuated during the experiment at 9.17 ± 0.18 at the high light intensity and 8.50 ± 0.22 at the low light intensity.

### Biochemical tissue analyses

Meristematic tissue from triplicate individual sporophytes were used for determination of DM (“Dry matter determination” section), carbon (C) and nitrogen (N) content (“Determination of C and N content” section). The reproductive status of the individual sporophytes used for biochemical tissue analyses was not recorded.

#### Dry matter determination

After freeze-drying, samples were weighed and DM was calculated as DW/FW × 100%.

#### Determination of C and N content

C and N content of the dry algae material was analysed using an elemental analyser (Roboprep C/N, Europa Scientific Ltd., UK) in line with a triple collector isotopic ratio mass spectrometer (Tracermass, Europa Scientific Ltd., UK). The tissue content of N (% of DM) was used as a proxy for the availability of inorganic N during growth.

#### Determination of monosaccharide composition

The determination of monosaccharide content in the environmental and experimental samples was carried out at two different labs using different protocols. All monosaccharides were expressed as dehydrated monomer values in % of DM.

##### Monosaccharide composition—seasonal variation in natural populations

The dried material was ground by vibrating disc milling to pass a 100-μm sieve. Three whole seaweed individuals were pooled proportionally to their weight into a single representative sample. Analysis of monosaccharides was performed as described in Manns et al. (2014): a two-step sulphuric acid treatment was applied in triplicate for each population and species (each replicate comprising three sporophytes as described above). Following monomeric sugars, sugar alcohol mannitol and uronic acids in the sulphuric acid hydrolysates were separated by high-performance anion exchange chromatography with pulsed amperometric detection (HPAEC-PAD).

##### Monosaccharide composition—lab experiments

The content of specific monosaccharides (glucose, xylose, mannose, galactose, fucose and mannitol) was determined in principle after a two-step sulphuric acid hydrolysis, i.e. 60 min treatment with 72% H_2_SO_4_ at 30 °C followed by 60 min hydrolysis in 4% H_2_SO_4_ at 121 °C. The pH of the hydrolysate was adjusted to the range of 4∼6 by CaCO_3_ and the hydrolysate was filtrated by 0.2 μm before being analysed using high-performance liquid chromatography (HPLC).

Contents of the specific monosaccharides glucose, xylose, mannose, galactose, fucose and mannitol were quantified by HPLC using a refractive index detector equipped with an Aminex HPX-87P column (Bio-Rad, USA) running at 84 °C with Milli-Q as eluent and a flow rate of 0.6 mL min^−1^.

The tissue content of cell wall and storage polysaccharides were estimated by summing of the individual monosaccharides and sugar acids constituting the different polysaccharides: crude fucoidan = fucose + galactose + mannose + xylose + glucuronic acid + arabinose. This will underestimate the true content of crude fucoidans, since the content of sulphates was not determined and included. Laminarin = glucose, minus glucose deriving from cellulose. The glucose fraction deriving from cellulose was estimated as the lowest seasonal glucose content observed for each species (Black 1950). Alginate = mannuronic + guluronic acid (with mannuronic acid quantified as galacturonic acid equivalents) (Manns et al. 2014, 2017). Storage carbohydrates: laminarin + mannitol.

### Statistical analyses

First, we tested the correlations between the key environmental factors used as independent variables: light, salinity, temperature, environmental ortho-P concentration, tissue N content (NDM) and REI, using Pearson correlation. Some of the independent variables showed substantial correlations, and hence, caution with regard to co-linearity was required.

For testing the effects of light, salinity, REI and NDM on the tissue contents of crude fucoidan and storage carbohydrates in the field samples, we used general linear models, which include multiple independent variables. For the experimental data, the models contained fewer variables: the model for experiment 1 only contained one variable, salinity, which had three discrete levels. Next, tests for pairwise differences were made using least square means. In experiment 2, we applied a model with two variables, light and tissue N content, and both variables were treated as a continuous variables. The tests were made using PROC MIXED or PROC GLM in SAS ver 9.3 (SAS Institute, USA). The residuals followed assumptions regarding normality and variance homogeneity. Although the relations may not be linear in the whole range of possible independent variables, we assumed a linear relation as this could describe the relation within the limited range of the natural concentrations or measures of other variables. More complex relations would require a larger range of values for the independent variables in this study.

Simple linear regressions were carried out for testing the correlations between the tissue contents of crude fucoidan and fucose and the ratio between crude fucoidan and alginate in the cell wall. For simple linear regressions and *t* tests, we used JMP, Version 12.1.0. (SAS Institute Inc., USA). Unless specified in the text, a significance level of 0.05 was applied.

## Results

### Seasonal variation in environmental factors at the sampling locations

**Fig. 2:**
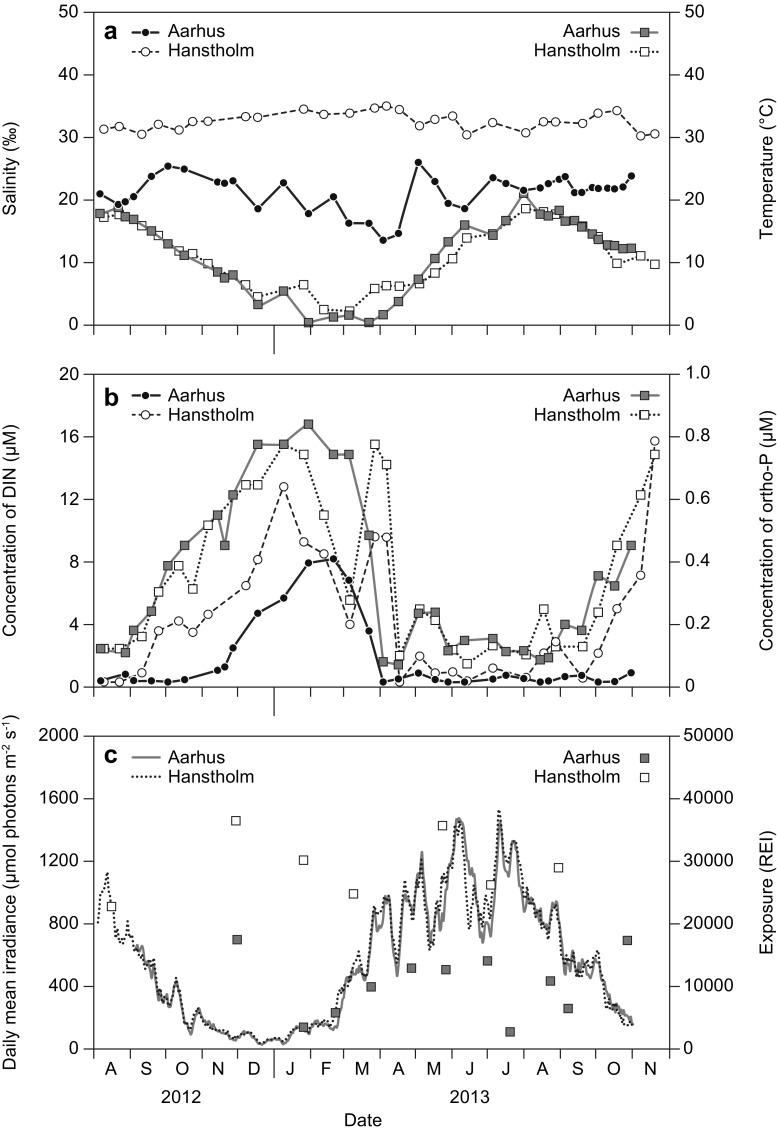
Seasonal variation of environmental factors at Aarhus (Kattegat) and Hanstholm (North Sea): **a** salinity and temperature; **b** inorganic nutrient concentrations, DIN and ortho-P; and **c** irradiance and exposure (relative exposure index (*REI*)). Data derived from the Danish National Marine Monitoring programme

The growth environment differed in some respects between the two sampling locations. The most pronounced differences were observed regarding salinity and exposure. At Hanstholm, in the North Sea, the salinity ranged between 30 and 35, with the highest during winter and the lowest during summer (Fig. 2a). At Aarhus, in Kattegat, the salinity fluctuated between 13 and 26 with the major period of low salinity in winter and spring. Marked fluctuations were here observed on a small temporal scale, most pronounced in May.

The fluctuations in water temperature over the year were similar between the two sites, ranging from 0 to 3 °C in winter to 18–22 °C in summer (Fig. 2a).

The dissolved inorganic nitrogen (DIN) concentrations ranged between 0 and 2 μM DIN during summer at both locations, and up to 8–16 μM DIN in the winter, with the highest at Hanstholm (Fig. 2b). The concentrations of ortho-P followed the fluctuations of DIN with summer concentrations of 0.1–0.3 μM ortho-P and winter concentrations of up to 0.8 μM ortho-P (Fig. 2b).

The incoming light over the year was similar at the two locations (Fig. 2c) ranging from a daily average minimum of 50–100 μmol photons m^−2^ s^−1^ in winter to a daily average summer maximum of up to 1500 μmol photons m^−2^ s^−1^.

The degree of exposure differed significantly between the two sites: Hanstholm, facing west at the North Sea coast, experienced exposure equivalent to an average REI of 30,794 ± 2018 over the sampling period, whereas Aarhus, facing east to the Kattegat, only experienced a third of the west coast exposure, with an average REI of 10,328 ± 1421 (*t* test, *p* = 0.004). At Hanstholm, the degree of exposure was highest in early winter and during a period in late May, whereas in Aarhus, the degree of exposure was high in late autumn and during spring and lowest during late winter and summer.

The environmental factors were intercorrelated in various respects and the correlations differed between the two sampling sites (Tables 2 and 3): in Aarhus, temperature and salinity were positively correlated, whereas at Hanstholm, they were negatively correlated. At Hanstholm, salinity was positively correlated to the concentration of inorganic nutrients (DIN and ortho-P). In Aarhus, DIN and ortho-P did not correlate to salinity, but both were inversely correlated to irradiance. At both locations, positive correlations were found between the concentrations of DIN and ortho-P, and negative correlations were observed between irradiance and ortho-P concentrations.

**Table 2:** Pearson correlation coefficients of key environmental parameters at Aarhus (Baltic Sea)

Aarhus (*n* = 11)	REI	PAR	DIN	Ortho-P	Temperature	NDM (SL)	NDM (LD)
Salinity	0.096 0.780	−0.089 0.796	−0.302 0.366	−0.148 0.664	*0.751* *0.008*	−0.373 0.258	−0.508 0.110
REI		−0.136 0.670	0.476 0.139	0.161 0.635	0.129 0.706	−0.188 0.579	−0.328 0.325
PAR			*−0.672* *0.024*	*−0.896* *<0.001*	0.507 0.111	*−0.763* *0.006*	−0.583 0.060
DIN				*0.894* *<0.001*	*−0.772* *0.005*	*0.921* *<0.001*	*0.854* *<0.001*
Ortho-P					*−0.716* *0.013*	*0.894* *<0.001*	*0.712* *0.013*
Temperature						*−0.857* *<0.001*	*−0.826* *0.002*

Two numbers are given for each correlation: the top number gives the correlation coefficient, bottom number the probability (*p* value). Numbers in italics indicate significant correlations (*p* < 0.05)

*REI* relative exposure index, *PAR* photosynthetic active radiation (μmol photons m^−2^ s^−1^), *DIN* dissolved inorganic nitrogen (μM), *ortho-P* ortho-phosphate (μM); water temperature (°C) and tissue nitrogen content (*NDM*) of *Saccharina latissima* (*SL*) and *Laminaria digitata* (*LD*) given as percentage of DM

**Table 3:** Pearson correlation coefficients of key environmental parameters at Hanstholm (North Sea)

Hanstholm (*n* = 8)	REI	PAR	DIN	Ortho-P	Temperature	NDM (LD)
Salinity	0.589 0.125	−0.462 0.249	*0.850* *0.008*	*0.779* *0.023*	*−0.820* *0.013*	*0.981* *<0.001*
REI		−0.212 0.615	0.588 0.125	0.655 0.078	−0.383 0.349	0.287 0.533
PAR			−0.641 0.087	*−0.704* *0.023*	0.640 0.09	−0.348 0.445
DIN				*0.953* *<0.001*	−0.623 0.099	*0.776* *0.040*
Ortho-P					−0.643 0.086	0.724 0.065
Temperature						*−0.830* *0.021*

Two numbers are given for each correlation: the top number gives the correlation coefficient, bottom number the probability (*p* value). Numbers in italics indicate significant correlations (*p* < 0.05)

*REI* relative exposure index, *PAR* photosynthetic active radiation (μmol photons m^−2^ s^−1^), *DIN* dissolved inorganic nitrogen (μM), *ortho-P* ortho-phosphate (μM); water temperature (°C) and tissue nitrogen content (*NDM*) of *Laminaria digitata* (*LD*) given as percentage of DM

### Seasonal variation in tissue composition

#### Crude fucoidan content

**Fig. 3:**
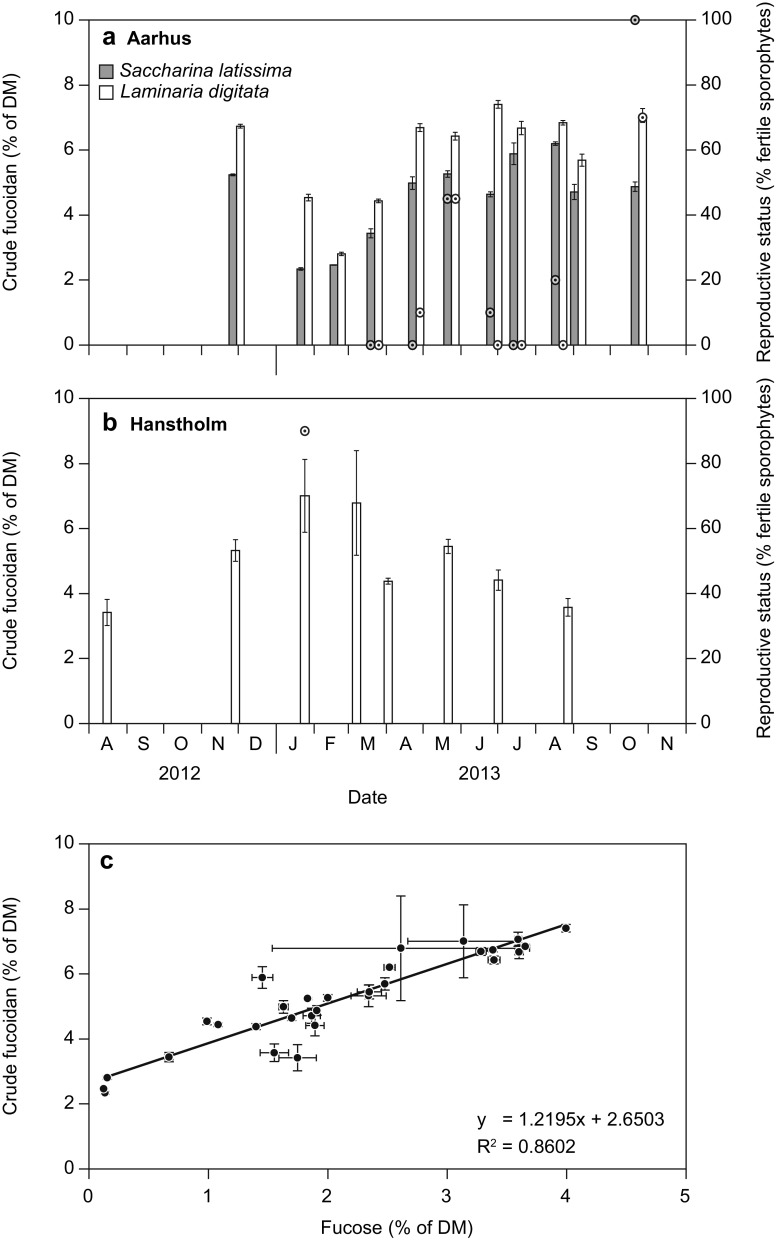
Seasonal variation in the tissue content of crude fucoidan and reproductive status (*circles*) in **a** *Saccharina latissima* and *Laminaria digitata* from Kattegat; **b** *Laminaria digitata* from the North Sea; and **c** relation between the tissue content of fucose and the calculated crude fucoidan content (*R* ^2^ = 0.86). Data represent average ± SE, *n* = 3

The content of crude fucoidan was on average 21% higher in *L. digitata* than in *S. latissima*: 5.04 ± 0.29% of DM and 5.94 ± 0.24% of DM in *L. digitata* in Hanstholm and Aarhus, respectively, compared to 4.55 ± 0.22% of DM in *S. latissima* in Aarhus (*t* test, *p* > 0.0001, *df* = 10) (Fig. 3). In Aarhus, where the two species co-exist, the content of crude fucoidan in *L. digitata* was 31% higher than in *S. latissima* (*t* test, *p* > 0.0001, *df* = 10). Within *L. digitata*, the content of crude fucoidan was 18% higher in the Kattegat population, than in the North Sea population. This difference was not statistically significant. The tissue content of crude fucoidan varied over the year by a factor of 2 in *L. digitata* at Hanstholm and a factor of 2.6 for both species in Aarhus. The pattern of the seasonal variation—summer maximum and winter minimum—was parallel in the two species from the Aarhus populations: the maximum and minimum tissue concentrations of crude fucoidan in *L. digitata* of 7.40 ± 0.12 and 2.81 ± 0.05% of DM were observed in late June and late February, respectively. For *S. latissima*, the maximum and minimum contents of 6.20 ± 0.06 and 2.34 ± 0.04% of DM were observed in late August and late January, respectively. In Aarhus, a high prevalence of fertile sporophytes of both species was observed in May and October, but the peak in fertility did not coincide with maximal fucoidan contents (Fig. 3a). The opposite seasonal pattern was observed in the Hanstholm population of *L. digitata*, where the minimum fucoidan concentration (3.42 ± 0.40% of DM) was observed in mid-August (2012), and the maximum concentration of 7.00 ± 1.12 was observed in late January, coinciding with the single observation of high prevalence of fertile sporophytes (Fig. 3b).

Across species and populations, a significant linear relation was found between the content of crude fucoidan and the content of the monosaccharide fucose (linear regression: *p* < 0.0001, *R* ^2^ = 0.854) (Fig. 3c).

**Fig. 4:**
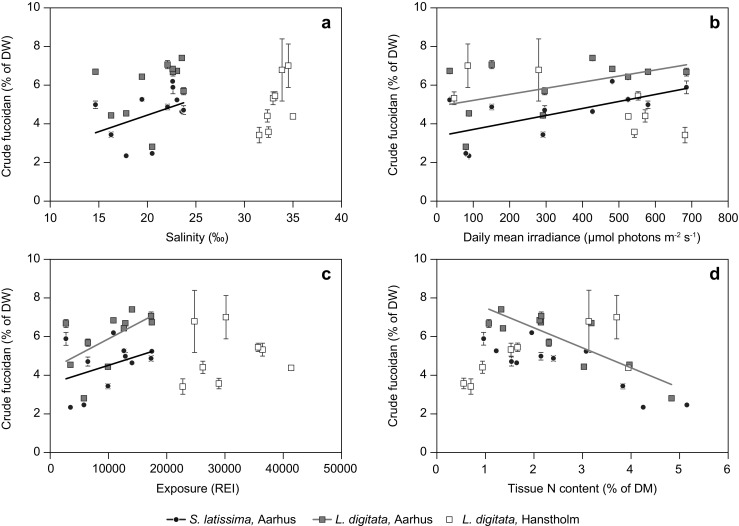
The tissue content of crude fucoidan as a function of single environmental factors: **a** salinity; **b** irradiance; **c** exposure (relative exposure index (*REI*)); **d** tissue N content (% of DM). Data represent average ± SE, *n* = 3. Statistics are given in Table 4. Only significant relations are indicated by *lines*

The relations between the tissue content of crude fucoidan and the four environmental factors varied between the populations from the two locations, and to a minor degree also between the two species at the same location (Aarhus). Regarding salinity (Fig. 4a, Table 4), pronounced differences in salinity ranges were observed between the two locations. The tissue content of crude fucoidan related positively to salinity in *S. latissima* in Aarhus, whereas this relation was not found for *L. digitata* at any of the two sites. For irradiance (Fig. 4b, Table 4), a positive and significant relation between crude fucoidan content and irradiance was observed in both kelp species of the Aarhus populations, but not in Hanstholm. For exposure (Fig. 4c, Table 4), the tissue content of crude fucoidan and the degree of exposure showed significant positive linear relation for both species in Aarhus, whereas no relation was observed at Hanstholm. Finally, regarding N availability (estimated as tissue N content) (Fig. 4d, Table 4), a significant negative relation between the tissue contents of N and crude fucoidan appeared only within the *L. digitata* population in Aarhus, whereas the overall picture across sites and species indicated more of a bell-shaped curve with maximal crude fucoidan contents related to tissue N contents in the range between 1.5 and 2.5% N (% DM).

**Table 4:** The impact of abiotic environmental factors on the tissue content of crude fucoidan of the two kelp species, *S. latissima* and *L. digitata*, sampled at two different locations in Denmark: Aarhus (Baltic Sea, Kattegat) and Hanstholm (North Sea). Results of the statistical analyses, testing by general linear models, including the following variables: salinity, irradiance, degree of exposure and tissue N content. *p* values < 0.05, indicating significant relation to the specific environmental parameter, are marked in italics

Species	Predictor	*F* value	*df*	Estimate	*p* value
*S. latissima* (Aarhus)	*Salinity*	*9.80*	*28*	*0.150*	*0.004*
	*Exposure (REI)*	*11.69*	*28*	*9.4 E-05*	*0.002*
	*PAR (μmol photons m* ^*−2*^ *s* ^*−1*^ *)*	*10.59*	*28*	*0.003*	*0.003*
	Tissue N (% of DM)	0.87	28	−0.168	0.360
*L. digitata* (Aarhus)	Salinity	0.32	28	0.028	0.576
	*Exposure (REI)*	*17.53*	*28*	*0.0001*	*<0.001*
	*PAR (μmol photons m* ^*−2*^ *s* ^*−1*^ *)*	*4.80*	*28*	*0.002*	*0.037*
	*Tissue N (% of DM)*	*13.25*	*28*	*−0.762*	*0.001*
*L. digitata* (Hanstholm)	Salinity	0.18	16	−0.391	0.680
	Exposure (REI)	0.38	16	−4.0E-5	0.550
	PAR (μmol photons m^−2^ s^−1^)	2.19	16	−0.002	0.159
	Tissue N (% of DM)	1.44	16	0.834	0.247

#### Structural and storage carbohydrates

In *S. latissima* at Aarhus, the content of storage carbohydrates ranged from 3% of DM in late January to 30% of DM in late September (data not shown, Manns et al. 2017). In the Aarhus population of *L. digitata*, the content of storage carbohydrate content ranged from 2% of DM in late February to 36% of DM in early September (data not shown, Manns et al. 2017). In the *L. digitata* population at Hanstholm, the storage carbohydrate content was considerably higher, ranging from a minimum of 10% of DM in early April to 52% of DM in mid-August 2012 (data not shown). The content of storage carbohydrates in August 2012 was higher than in August 2013, where a content of storage carbohydrates of 35% of DM was registered in late August.

**Fig. 5:**
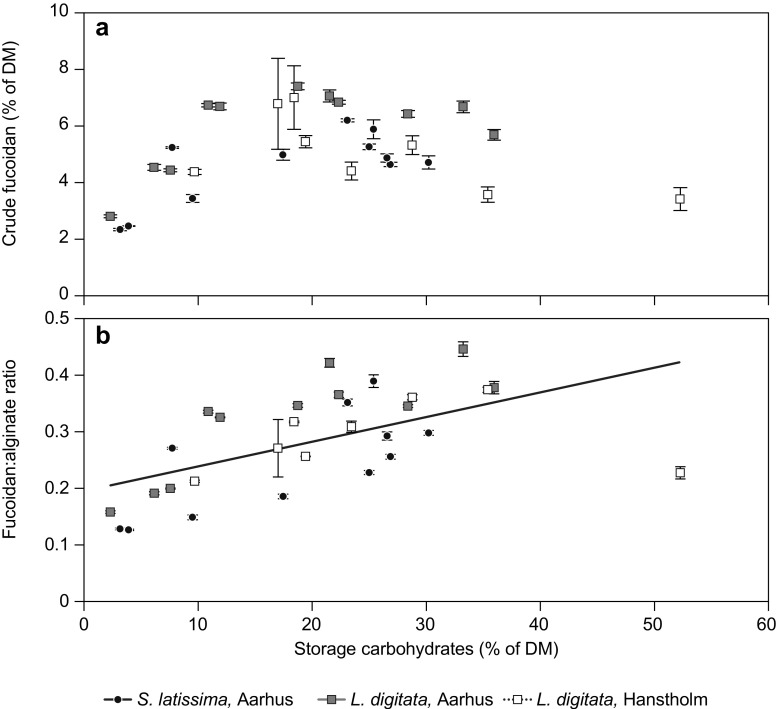
Relations between **a** the tissue content of crude fucoidan and storage carbohydrates (laminarin + mannitol); **b** the ratio between crude fucoidan and alginate in the cell wall, and the tissue content of storage carbohydrates. The significant linear correlation is indicated (*p* < 0.0001, *R* ^2^ = 0.326). Data represent average ± SE, *n* = 3

A bell-shaped relation was observed for the tissue content of crude fucoidan as a function of storage carbohydrates (Fig. 5a). Here a broad peak in crude fucoidan content was observed to coincide with approximately 20% storage carbohydrates of DM.

A positive linear correlation was found between the ratio of the two major structural carbohydrates in the cell wall, crude fucoidan and alginate, and the tissue content of storage carbohydrates (linear regression: *p* < 0.0001, *R* ^2^ = 0.326) (Fig. 5b). This ratio fluctuated by a factor of 3 over the year in a similar manner for the three populations: the lowest ratios of fucoidans to alginate (0.13–0.21) were observed during late winter and early spring, and the highest ratios were observed during late summer and autumn (0.37–0.45). The ratio of crude fucoidan to alginate was generally lower in *S. latissima*, compared to *L. digitata*.

The content of storage carbohydrates showed, as for crude fucoidan, different relations to environmental factors depending on species and location (Table S1, Fig. S1): in Aarhus, the content of storage carbohydrates related positively to salinity (only *L. digitata*) and negatively to tissue content of inorganic N (both species) and exposure (only *L. digitata*). No significant relations between tissue storage carbohydrate content and environmental factors were found for the Hanstholm population.

#### Tissue C and N

Generally, the fluctuations in the tissue contents of C and N were similar among the populations of the two different species at Aarhus. The tissue contents of C ranged between 26% of DM and 39% of DM in the Aarhus populations with a general minimum in spring and higher values in the summer/autumn period. The C content in the Hanstholm *L. digitata* population ranged between 27% of DM in late June and 40% of DM in late August (data not shown).

The tissue N content in all populations peaked in late winter with tissue content as high as 5.3% of DM in the Aarhus populations (data not shown). During late spring and summer, the N content decreased to minimum values of 0.6–2% of DM, with the lowest N contents and the longest period of low N content observed in the Hanstholm population.

At both locations and in both species, positive and significant correlations were found between the environmental concentrations of DIN and tissue N concentrations (Tables 2 and 3).

### Experiments

The content of crude fucoidan of the fronds sampled and used for experimental work was similar to the content of crude fucoidan observed in the natural populations sampled at the given season (*S. latissima* in January and *L. digitata* in June).

**Fig. 6:**
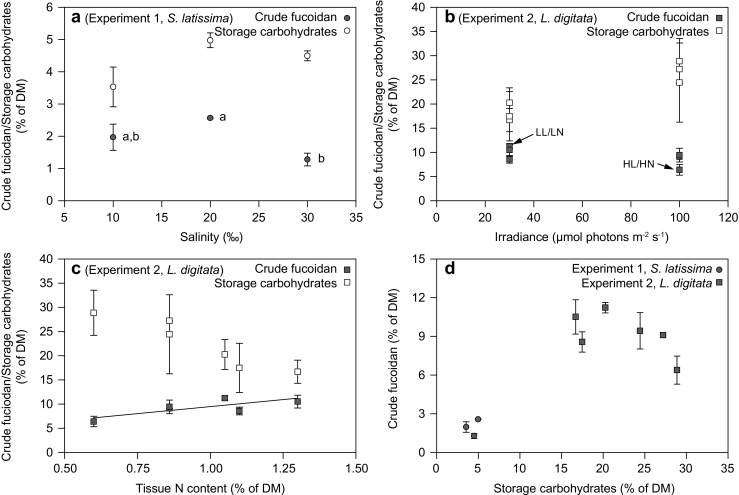
Results from experiments 1 and 2, showing the relation between crude fucoidan or storage carbohydrates (laminarin + mannitol) and salinity, irradiance or tissue N content (**a**–**c**) or the relation between crude fucoidan and storage carbohydrates (**d**). Data represent average ± SE, *n* = 2: **a** salinity (experiment 1, *S. latissima*). *Letters a* and *b* indicate significant differences between crude fucoidan contents. Contents of storage carbohydrates were not significantly different between salinity treatments; **b** irradiance (experiment 2, *L. digitata*). The two specific experimental treatments: high light and high nutrient concentration (HL/HN) and low light and low nutrient concentration (LL/LN) are indicated; **c** tissue N content (% of DM) (experiment 2, *L. digitata*). The *solid line* indicates the significant relation between crude fucoidan content and tissue N content (GLM *F*_2,8_ = 10.16, *E* = 5.12, *p* = 0.013); and **d** relations between crude fucoidan content and content of storage carbohydrates from both experiments

In experiment 1 (*S. latissima*, salinity), the content of crude fucoidan in the individual sporophytes of *S. latissima* peaked at a salinity of 20 (2.57 ± 0.03% of DM) (GLM *F*_2,8_ = 6.28, *p* = 0.023) (Fig. 6a). The crude fucoidan content at a salinity of 20 only differed significantly from that at a salinity of 30 (least square means, *p* = 0.024), and not from the crude fucoidan content at a salinity of 10 (least square means, *p* = 0.157). Also, the tissue content of storage carbohydrates peaked at a salinity of 20 (4.98 ± 0.23% of DM); however, no significant differences in the content of storage carbohydrates were found between any of the salinity treatments (Fig. 6a).

In experiment 2 (*L. digitata*, light and DIN), the tissue content of crude fucoidan in *L. digitata* ranged from 6.38 ± 1.08% of DM in the sporophytes exposed to the high light and high DIN treatment to 11.22 ± 0.41% of DM in the low light and low DIN treatment (Fig. 6b). The fucoidan content was positively related to the tissue N content (GLM *F*_2,8_ = 10.16, *E* = 5.12, *p* = 0.013), but did not relate significantly to light (GLM *F*_2,8_ = 0.59, *E* = 0.01, *p* = 0.571). The content of storage carbohydrates was higher in the sporophytes exposed to the high light intensity (Fig. 6b) and tended to decrease with increasing tissue N content (Fig. 6c). However, the storage carbohydrate content did not relate significantly to neither light nor tissue N content (GLM *F*_2,8_ = 1.44, *E* = 0.09, *p* = 0.265 and GLM *F*_2,8_ = 0.37, *E* = −4.55, *p* = 0.560, respectively).

The results of both experiments fit into the bell-shaped relation between fucoidan and storage carbohydrates described by the results from the natural populations, with fucoidan content peaking at approximately 20% storage carbohydrates (Figs. 5a and 6d).

## Discussion

### Seasonal variation in crude fucoidan content

The tissue contents of crude fucoidan of *L. digitata* found in this study were higher than, or within the range of, the concentrations of up to 5.5% of DM previously reported for this species (Black 1954; MacArtain et al. 2007) and higher than the concentrations found in *S. latissima* at the same location. The crude fucoidan concentrations of *S. latissima* were within the concentration range of 1.8–8.8% of DM described by others (Black 1954; Obluchinskaya 2008; Ehrig and Alban 2015).

The seasonal patterns of late summer maximum fucoidan contents in *S. latissima* and *L. digitata* at Aarhus were in agreement with the findings of most other studies (Honya et al. 1999; Obluchinskaya 2008; Skriptsova et al. 2010; Ehrig and Alban 2015; Skriptsova 2016), as well as with the seasonal pattern of L-fucose content in *L. digitata* described by Black (1954). The contrasting seasonal pattern of fucoidan content of *L. digitata* at Hanstholm was more in agreement with the summer minimum and a winter maximum tissue content of crude fucoidan that was described for North Atlantic *S. latissima* (Black 1954). The difference in seasonal pattern of fucoidan content between the Hanstholm and Aarhus populations of *L. digitata* was not reported for other cellular components (alginic acid, mannitol and glucose, protein, ash) (Manns et al. 2017). However, considerably higher contents of glucose in autumn and alginate in spring were reported from the Hanstholm population, as compared to the Aarhus population (Manns et al. 2017). The peak of crude fucoidan content in *L. digitata* in Hanstholm was observed at the same time as high prevalence of reproductive tissue in the sporophytes was observed, and this could indicate a coupling between high fucoidan content and reproduction in this species, as has been shown for *Alaria* species, *S. japonica* and *U. pinnitifida* (Skriptsova et al. 2010, 2012; Vishchuk et al. 2012). In the Aarhus population of *L. digitata* (and *S. latissima*) however, reproductive status was not coupled to fucoidan content, and it could appear that the two *L. digitata* populations had a different seasonal timing of reproduction. However, since only one observation on fertility was registered from Hanstholm, and since observations on reproductive status were never made from both *L. digitata* populations in the same months, no conclusions can be made on this basis and further investigations are needed.

### Effects of environmental factors

Looking into the single environmental factors to explain the patterns of seasonal variation in fucoidan content, the results of this study indicated that the cell wall content of fucoidan in the two kelp species did appear to respond directly to certain environmental factors/stressors; however, the responses to single environmental factors varied between species and between populations of the same species and were not consistent on a general basis. The fronds used in the experimental work were representable for the natural populations, regarding size, age and fucoidan content of the fronds (Skriptsova 2016, Zvyagintseva et al. 2003).

First addressing salinity, the results to some extent support that the crude fucoidan content would respond to osmotic stress to the cell wall, by more fucoidan being synthesized for strengthening the cell wall by cross-linking matrix cellulose microfibrils (Deniaud-Bouet et al. 2014). However, the results obtained for *S. latissima* from the field and the lab were not fully in agreement: the natural population of *S. latissima* showed significantly increased content of fucoidan with increasing salinities in the salinity range from 15 to 25‰ (Fig. 4a, Table 4), indicating a cellular response to osmotic stress, as previously suggested by Ehrig and Alban (2015) comparing the fucoidan content of North Atlantic and Baltic populations of *S. latissima*. When studying the response to salinity in fucoidan content in *L. digitata*, it appeared as if a positive relation could indicate the same mechanism in both species within their natural salinity range; however, in *L. digitata*, this trend was not significant (Fig. 4a, Table 4). The results from *S. latissima* in experiment 1 were in agreement with this in the lower end of the salinity range, with the crude fucoidan content increasing with increasing salinities from 10 to 20‰. However, the significant decrease in fucoidan content at salinities exceeding the natural range experienced by the Aarhus population (30‰) could indicate an adaptation to the local environment, with salinity stress above and below average being met by decreased contents of fucoidan in the cell walls of *S. latissima*, as lower fucoidan content was observed at salinities that were both lower (10‰) and higher (30‰) than the average salinity of approximately 20‰ experienced by the Aarhus population (Fig. 6a). Regarding the effect of mechanical stress via exposure, both species from Aarhus responded to increased exposure with significantly increasing tissue contents of fucoidan, which was the opposite as found by Black (1954); however, in Hanstholm, where the degree of exposure was three times higher than in Aarhus, there was no significant relation between REI and fucoidan content. This could indicate a non-linear relation between exposure and crude fucoidan, which may be linear and positive at lower fucoidan contents, however eventually approaching a physiologically maximal fucoidan content as a horizontal asymptote. It could also indicate a differentiation between ecotypes of *L. digitata* to two very different environments.

With regards to light, Holtkamp (2009) suggested, but did not demonstrate, that the relative tissue content of fucoidan would relate positively to irradiance as a response to increased oxidative stress caused by high light and UV radiation. Our results from the natural populations in Aarhus could support this theory, as both species here showed a positive relation between fucoidan content and irradiance (Fig. 4b, Table 4). The positive relation between temperature and content of fucoidan in *S. japonica* (Skriptsova 2016) may also indirectly be in support of this, as generally high irradiance will be a proxy for higher surface water temperature. A more simple explanation to the positive relation between light and crude fucoidan could be that synthesis of fucoidan is stimulated through increased production of primary photosynthetic products (Michel et al. 2010). However, no significant relation between light and fucoidan content was found in the natural population of *L. digitata* in Hanstholm (Fig. 4b, Table 4), indicating again a differentiation between the two populations of *L. digitata*.

In addition to the direct effects of environmental factors, we addressed the hypothesis that the relative tissue content of fucoidan could be regulated by the relative content of storage polysaccharides in the sense that the cell wall would “stretch”, as the cells expand to accommodate the accumulated storage polysaccharides. Here we found that the general response of tissue fucoidan to storage polysaccharides was not linear, but formed a bell shape function with maximum fucoidan content at intermediate contents of storage polysaccharides (∼20% of DM). This could be interpreted as a two-phase response, where the second phase is in support of the hypothesis (Figs. 5a and 6d): in phase 1, the tissue contents of storage carbohydrates and fucoidan increase as a response to increased irradiance. In this phase, the synthesis of both cell wall carbohydrates and storage carbohydrates is taking place. In phase 2, growth (defined as cell division) of the sporophyte ceases due to exhaustion of intracellular nitrogen reserves, but accumulation of storage carbohydrates continues (Chapman and Craigie 1977). In this phase, only storage carbohydrates are synthesized, causing the relative content of other cellular components to decrease proportionally. Hence, in phase 2, an inverse relation between storage carbohydrates and fucoidan would be observed. The two-phase interpretation is supported by the shift between the two phases being observed at a tissue concentration of storage carbohydrates of approximately 20% of DM (Figs. 5a and 6d): this concentration coincides with a tissue N content in the range of 2% of DM, which is comparable to the N content critical for growth, N_C_, defined at 1.88 for *S. latissima* (Chapman et al. 1978), and at 1.7 for brown algae in general (Pedersen and Borum 1997). This is further supported by the significant relations between crude fucoidan content and tissue N content in Aarhus population of *L. digitata*, that show a positive relation at tissue N concentrations <1.5 (experiment 2) (Fig. 6c), and a negative relation at tissue N contents >1.5 (natural populations) (Fig. 4d). Thus, the tissue content of fucoidan may be controlled in a two-phase manner by the tissue content of storage carbohydrates, with the tissue content of N as the trigger of phase shift. As the content of storage carbohydrates increases, the cell wall ratio between fucoidan and alginate increases (Fig. 5b), indicating an increased relative content of fucoidan in the cell wall. To what extent this could increase the strength of the cell wall as it stretches (Deniaud-Bouet et al. 2014) or indicates a need for a constant content of fucoidan relative to cell surface in order to resist viral or bacterial attacks to the cell wall (Mandal et al. 2007; Wijesekara et al. 2011) needs further investigations.

### Implications for harvest and cultivation

Our study confirmed that adjusting timing of biomass harvest to the time of natural maximal fucoidan will increase the potential harvest yield of fucoidan in kelps—in this study, by a factor of 2–2.6. Most often, maximal fucoidan contents are described in summer (Honya et al. 1999; Skriptsova et al. 2010; Skriptsova 2016); in this study, however, we observed different seasonal peaks of fucoidan content between populations: at Hanstholm in the North Sea, the fucoidan content of *L. digitata* peaked in late winter coinciding with high alginic acid content (Manns et al. 2017), whereas the fucoidan content of the Kattegat populations of *L. digitata* and *S. latissima* both peaked in late summer. Thus, generalized recommendations for harvest time are difficult to make. Maximal tissue content of fucoidan did not always coincide with maximal tissue content of other cell wall or storage carbohydrates, implicating an economical trade-off, if the harvested kelp biomass is to be exploited in a sequential or cascading biorefinery concept with focus on fucoidan as well as alginate or energy production (Hou et al. 2015). However, viewed in the perspective of areal yield of fucoidan and not relative tissue content, it may be optimal to harvest at the peak of storage carbohydrate content, since the areal yield of crude fucoidan may remain constant while the areal yield of storage carbohydrates increases, driving the relative decrease in tissue fucoidan content. This remains to be documented in large-scale cultivation, and the optimal harvest time will thus depend on the design of the biorefinery and the efficiency of its processes for carbohydrate extractions.

In contrast to what is previously described in literature, we found that the content of fucoidan in *L. digitata* was higher than in *S. latissima*. *Saccharina latissima* is, however, at present the most commonly cultivated species of the two in Europe and North America (Kerrison et al. 2015). Despite having lower fucoidan concentrations, the fucoidan content of the investigated population of *S. latissima* appeared to respond directly to environmental factors, indicating a potential for manipulating the fucoidan content of this species through the cultivation process: fucoidan contents increased by 25% (from 4 to 5% of DM in natural populations and from 2 to 2.5% of DM in experiment 1) with salinity changes from 15 to 25 and 10 to 20, respectively. Thus, theoretically, positioning of farms at salinities between 20 and 25, or even a reposition of a cultivation structure across a pycnocline from lower salinity to higher salinity, could increase the harvest yield of fucoidan. However, shifting the sporophytes to a position lower in the water column would at the same time implicate a lower irradiance as well as a decrease in wave exposure, both of which would tend to counteract the increase in fucoidan content according to our findings. Thus, in a practical perspective, it remains questionable, if such operations would be cost-efficient.

The two Danish populations of *L. digitata* demonstrated differences in seasonal pattern of fucoidan content, which supports previous reports of differences in biochemical composition of kelps in relation to the salinity gradient prevailing in Danish waters (Nielsen et al. 2016a). The further difference in response to environmental factors indicated a differential adaptation to the different growth environments experienced by the two populations—the high saline, exposed environment in the North Sea versus the more sheltered, less saline Kattegat. In *S. latissima*, the populations in Kattegat have a lower genetic diversity, as compared to North Sea populations, potentially reflecting an adaptation to the less saline environment (Nielsen et al. 2016b). The selective pressure exerted by the salinity gradient from the North Sea to the Baltic is also reflected by a general decrease in macroalgae species diversity in the North Sea-Baltic transitions zone (Middelboe et al. 1997). Genetic differentiation of *L. digitata* populations over distances <10 km has been described in the English Channel despite connectivity of the populations (Billot et al. 2003), demonstrating the limited range of dispersal of kelp propagules. Thus, a genetic differentiation between the populations of *L. digitata* in Hanstholm and Aarhus is a potential explanation for the differences observed. In a cultivation perspective, as well as in an ecological perspective, this emphasizes the need for a mapping of the genetic diversity of European kelps, as well as for investigating the biochemical and physiological consequences of the specific genetic differentiations.

## Conclusion

The crude fucoidan content, and potential harvest yield, in the studied natural populations of *S. latissima* and *L. digitata* varied by a factor of 2–2.6 over the year. The different seasonal variations in fucoidan content and the different responses to environmental factors between the two geographically separated populations of *L. digitata* indicate a genetic differentiation between the populations as described for *S. latissima*. The fucoidan content of the Kattegat population of *S. latissima* related positively to salinity, irradiance and exposure. In both species, the highest content of fucoidan was observed to coincide with intermediate contents of storage polysaccharides (∼20% of DM), as sporophytes exhausted the tissue N content below the concentration critical for growth (N_C_). The ambiguous direct response to the single environmental factors salinity, exposure and irradiance, between and among species, complicates prospective directions for manipulating an increased content of fucoidan in a cultivation scenario and calls for investigations of the genetic diversity of European kelp ecotypes and their physiological and ecological characteristics.

## Electronic supplementary material

Fig. S1 The tissue content of storage carbohydrates (laminarin + mannitol) as a function of environmental factors, **a**) salinity; **b**) irradiance; **c**) exposure (relative exposure index (REI)); **d**) tissue N content (% of DM). Data represent average ± SE, *n* = 3. Statistics are given in Table S1. (JPEG 51 kb)

High resolution image (EPS 2497 kb)

Table S1 (DOCX 17 kb)
