# Supplementary figures and images for: Crude fucoidan content in two North Atlantic kelp species, Saccharina latissima and Laminaria digitata—seasonal variation and impact of environmental factors
Source: J Appl Phycol. 2017 Jul 5;29(6):3121–37. doi: 10.1007/s10811-017-1204-5 (PMC5705760; doi:10.1007/s10811-017-1204-5)

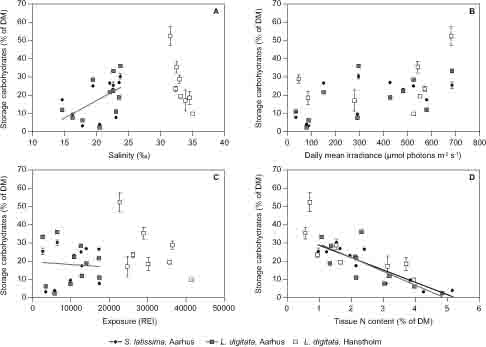

Supplement: Supplementary file 1 — The tissue content of storage carbohydrates (laminarin + mannitol) as a function of environmental factors, a) salinity; b) irradiance; c) exposure (relative exposure index (REI)); d) tissue N content (% of DM). Data represent average ± SE, n = 3. Statistics are given in Table S1. (JPEG 51 kb) [file 10811_2017_1204_Fig7_ESM.jpg]
