# Supplementary material for: Crude fucoidan content in two North Atlantic kelp species, Saccharina latissima and Laminaria digitata—seasonal variation and impact of environmental factors
Source: J Appl Phycol. 2017 Jul 5;29(6):3121–37. doi: 10.1007/s10811-017-1204-5 (PMC5705760; doi:10.1007/s10811-017-1204-5)
Supplement: Supplementary file 3 — (DOCX 17 kb) [file 10811_2017_1204_MOESM2_ESM.docx]

Table S1. The impact of abiotic environmental factors on the tissue content of storage carbohydrates of the two kelp species, *S. latissima* and *L. digitata*, sampled at two different locations in Denmark: Aarhus (Baltic Sea, Kattegat) and Hanstholm (North Sea). Results of the statistical analyses, testing by General Linear Models including the following variables: salinity, irradiance, degree of exposure and tissue N content. P-values <0.05, indicating significant relation to the specific environmental parameter, are marked in bold.

| **Species** | **Predictor** | **F-value** | **df** | **Estimate** | **P-value** |
| --- | --- | --- | --- | --- | --- |
| *S. latissima* (Aarhus) | Salinity | 3.58 | 28 | 0.666 | 0.069 |
|  | Exposure (REI) | 0.07 | 28 | -6E-05 | 0.786 |
|  | PAR (µmol photons m^-2^ s^-1^) | 0.21 | 28 | 0.003 | 0.649 |
|  | **Tissue N (% of DM)** | **17.73** | **28** | **-5.586** | **<0.001** |
| *L. digitata* (Aarhus) | **Salinity** | **4.44** | **28** | **1.026** | **0.044** |
|  | **Exposure (REI)** | **5.42** | **28** | **-0.0006** | **0.027** |
|  | PAR (µmol photons m^-2^ s^-1^) | 3.11 | 28 | 0.013 | 0.089 |
|  | **Tissue N (% of DM)** | **9.92** | **28** | **-6.407** | **0.004** |
| *L. digitata* (Hanstholm) | Salinity | 0.70 | 16 | -3.593 | 0.415 |
|  | Exposure (REI) | 0.11 | 16 | -0.0001 | 0.749 |
|  | PAR (µmol photons m^-2^ s^-1^) | 3.35 | 16 | -0.013 | 0.086 |
|  | Tissue N (% of DM) | 1.08 | 16 | -3.334 | 0.314 |
